# Impact of Exogenous Gonadotropin Stimulation on Circulatory and Follicular Fluid Cytokine Profiles

**DOI:** 10.1155/2014/218769

**Published:** 2014-11-30

**Authors:** N. Ellissa Baskind, Nicolas M. Orsi, Vinay Sharma

**Affiliations:** ^1^The Leeds Centre for Reproductive Medicine, Leeds Teaching Hospitals NHS Trust, Seacroft Hospital, York Road, LS14 6UH Leeds, UK; ^2^Women's Health Research Group, Leeds Institute of Cancer & Pathology, St James's University Hospital, Wellcome Trust Brenner Building, Beckett Street, LS9 7TF Leeds, UK

## Abstract

*Background*. The natural cycle is the prototype to which we aspire to emulate in assisted reproduction techniques. Increasing evidence is emerging that controlled ovarian hyperstimulation (COH) with exogenous gonadotropins may be detrimental to oogenesis, embryo quality, and endometrial receptivity. This research aimed at assessing the impact of COH on the intrafollicular milieu by comparing follicular fluid (FF) cytokine profiles during stimulated *in vitro* fertilization (IVF) and modified natural cycle (MNC) IVF. *Methods*. Ten women undergoing COH IVF and 10 matched women undergoing MNC IVF were recruited for this pilot study. 40 FF cytokine concentrations from individual follicles and plasma were measured by fluid-phase multiplex immunoassay. Demographic/cycle/cytokine data were compared and correlations between cytokines were computed. *Results*. No significant differences were found between COH and MNC groups for patient and cycle demographics, including outcome. Overall mean FF cytokine levels were higher in the MNC group for 29/40 cytokines, significantly so for leukaemia inhibitory factor and stromal cell-derived factor-1*α*. Furthermore, FF MNC cytokine correlations were significantly stronger than for COH data. *Conclusions*. These findings suggest that COH perturbs intrafollicular cytokine networks, in terms of both cytokine levels and their interrelationships. This may impact oocyte maturation/fertilization and embryo developmental competence.

## 1. Introduction

Controlled ovarian hyperstimulation (COH) with gonadotropins has improved success rates of* in vitro* fertilization (IVF) by increasing the number and opportunity for selection of embryos before transfer [1–3] as well as permitting the cryopreservation of supernumerary embryos for further fertility treatment [4, 5]. The basis of COH is to support the growth of multiple follicles to the preovulatory stage, a process achieved by bypassing physiological regulatory mechanisms. Urinary-derived or recombinant follicle stimulating hormone (FSH) is administered to increase serum concentrations above the threshold required for dominant follicle selection, thus enabling the entire cohort of recruited follicles to develop and attain preovulatory status [4]. Luteinising hormone (LH) is often coadministered although, following pituitary downregulation, this is not essential for follicular development as remnant basal LH levels are sufficient to stimulate the theca cells. Administration of a GnRH analogue (long protocol) or an antagonist (short protocol) that desensitizes the pituitary is primarily used to prevent premature LH surge as a consequence of supraphysiological serum oestradiol (E_2_) levels which, if it occurs, can lead to premature luteinisation and/or ovulation.

With few exceptions [6], the last two decades have witnessed a mounting body of evidence indicating that ovarian stimulation has a detrimental effect on oogenesis, embryo quality, and endometrial receptivity [7–13]. More specifically, Sharma et al. [7] and Pellicer et al. [14] demonstrated that retrieval of >10 oocytes per woman adversely affected their quality based on oocyte/embryo morphology, fertilization, and implantation rates. More recently van der Gaast et al. [15] found 13 oocytes to be the optimum number retrieved in order to achieve a pregnancy using a long protocol, above which there was a fall in pregnancy rates. An extreme example of the negative impact of COH is the excessively high number of poor quality oocytes seen in ovarian hyperstimulation syndrome (OHSS), which is putatively attributable to detrimental supraphysiological E_2_ levels [16]. These observations in humans are supported by a number of rodent studies that investigated the impact of exogenous gonadotropin stimulation on oocytes and demonstrated a delay in embryo development [17, 18]. It has been suggested that gonadotropin stimulation may affect oocyte maturation and the completion of meiosis, thus leading to an increased risk of having aneuploid oocytes and/or embryos [10, 19]. As such,* in vitro* maturation (IVM) has been proposed as an alternative strategy since it reduces exposure to exogenous gonadotropin stimulation, but the process itself introduces a host of other variables/complications (e.g., disruption of the meiotic spindle) that do not allow a fair comparison of these approaches to be made [20]. von Wolff et al. [21] recently demonstrated a varying endocrine follicular milieu together with the concentration of putative markers of oocyte quality, specifically anti-Müllerian hormone (AMH) between NC and COH FF, and suggest that this may be the cause for the lower oocyte quality following COH compared with naturally matured oocytes.

There has also been some concern that suppressed LH concentrations in the late follicular phase may be detrimental through downstream perturbations in follicular steroid synthesis. Consequently, stimulation protocols incorporating exogenous LH were developed, resulting in an increase in the percentage of diploid and good quality embryos obtained [22, 23]. By contrast, other investigators have reported a reduction in fertility and increased risk of miscarriage when incorporating exogenous LH into protocols [24, 25]. Such contradictory findings support the notion of a “LH window” below which E_2_ production is inadequate and above which LH may begin premature luteinisation and be detrimental to follicular development [26]. von Wolff et al. [21] postulate that the reduced levels of intrafollicular AMH they demonstrated following COH compared with NC may be attributed to LH suppression, resulting initially in low follicular androgen concentrations, and subsequently to low AMH production which in turn may be responsible for lower oocyte quality.

Natural cycle IVF (NC-IVF) has been proposed as an alternative treatment for older women and poor responders [27]. Indeed, there has been a resurgence of interest in NC-IVF for all patients in recent years because it avoids COH and its potential sequelae. Moreover, this also supports the international drive to reduce multiple pregnancies rates with elective single embryo transfer and to minimise complications such as OHSS [28–30]. Pelinck et al. [1] conducted a systematic review of 1,800 natural IVF cycles reported between 1989 and 2000 and concluded that NC-IVF has a pregnancy rate of less than 10% per cycle. More recent reports concur, presenting a similar 15.2% live birth rate per initiated cycle in all reported unstimulated NCs in women <35 years (*n* = 795) in the United States (2006-2007) [31].

A compromise between these approaches has been described: mild ovarian stimulation IVF. This method incorporates the use of low dose gonadotropin stimulation together with a gonadotropin releasing hormone (GnRH) antagonist aimed at generating fewer than eight oocytes per cycle [32]. The term modified natural cycle IVF (MNC-IVF) is applied when drugs (e.g., human chorionic gonadotropin (hCG)) are used to induce final oocyte maturation whereby a GnRH antagonist is administered during a spontaneous cycle to reduce the risk of cancellation [33] and/or where luteal support is provided.

During folliculogenesis, follicular fluid (FF) composition exhibits dynamic changes as individual follicular cell types respond to gonadotropins by secreting different hormones and cytokines [34, 35]. As growth factors regulating all stages of folliculogenesis, cytokines have been shown to govern the development/function of somatic cells and the oocyte as well as the composition of FF [36–42]. Given that oocyte quality influences subsequent embryo viability [43], it has been suggested that the disruption in the balance of these intrafollicular mediators following COH may influence cycle outcome [44–52]. The correct regulation of cytokine networks is essential to support normal physiology and this central role is underscored by the fact that inflammatory/immune dysfunctions underpin many pathological reproductive conditions, resulting in both local and systemic changes in cytokine profiles [53–55].

Studies have measured individual FF cytokines throughout the menstrual cycle. For example, the levels of interleukin- (IL-) 8, a chemotactic and angiogenic cytokine essential to folliculogenesis, have been found to rise from the midfollicular to the late follicular phase. These levels are comparable to those found during a COH cycle [56], implying that granulosa cell (GC) and theca cell (TC) IL-8 secretion is a true physiological phenomenon associated with follicular growth/maturation rather than resulting from gonadotropin stimulation.* In vitro* enhancement of IL-8 secretion by cultured GCs and TCs was evident following exposure to IL-1*α* and IL-1*β*, but not tumour necrosis factor- (TNF-) *α*, suggesting that IL-8 is both gonadotropin and cytokine-induced and may thus be involved in the hormonally regulated stages of folliculogenesis and ovulation [57].

Although cytokines are readily detected in FF, the complexity of their network regulation makes their study in isolation difficult to interpret. In view of their biological properties (pleiotropism, synergy, antagonism, functional redundancy, and differential sensitivity) [58–60], cytokines should ideally be investigated in terms of their interrelationships as much as in terms of their absolute concentrations. Whilst a recent study by Bersinger et al. [61] failed to demonstrate a difference in 13 FF cytokines between women undergoing NC and COH IVF, no specific attention was paid towards the complex interrelations within the cytokine networks. To date, there has been a paucity of studies focusing on minimal stimulation regimens and MNCs, and comparisons of isolated cytokine concentrations (e.g., vascular endothelial growth factor (VEGF) in COH and NC-IVF) have been inconsistent [62, 63]. Exogenous gonadotropins may disrupt intrafollicular cytokine networks, in turn affecting oocyte developmental potential. Therefore, this pilot study aimed at examining the impact of gonadotropins on the intrafollicular cytokine milieu in MNC and following COH cycles.

## 2. Materials and Methods 

### 2.1. Patient Recruitment and Sample Collection

From November 2008 to March 2009, ten women who required treatment with IVF/ICSI due to unexplained or male factor infertility aged 25–35 years with a body mass index (BMI) 19–30 were selected to undergo MNC-IVF/intracytoplasmic sperm injection (ICSI) at the Assisted Conception Unit, St James's University Hospital, Leeds, UK. These patients were matched with ten women undergoing COH-IVF/ICSI. Only nonsmokers and women who drank <6 units alcohol per week were included. They were required to be ovulatory (confirmed by transvaginal ultrasound scan (TVUSS), progesterone levels, or commercial LH surge kits within the preceding three months) and have a normal endocrine profile (early follicular phase FSH < 8.0 IU/L and E_2_ 50–200 pmol/L), a negative infection screen (including negative serum* Chlamydia* antigens), and normal pelvic anatomy confirmed by TVUSS and laparoscopy. Furthermore, they were required to have no risk factors for pelvic pathology (e.g., history of pelvic inflammatory disease, incomplete miscarriage, ectopic pregnancy, cervical dyskaryosis, and abdominal/pelvic/cervical surgery). Women with coexisting morbidity (e.g., autoimmune diseases, inflammatory conditions, and diabetes mellitus) and those taking regular medications were also excluded. The study protocol was approved by National Research Ethics Service, Leeds (East) Research Ethics Committee, and all participants provided written informed consent.

All women underwent a baseline TVUSS (ALOKA SSSD 1700) in the early follicular phase. In the MNC cohort, a TVUSS assessment was performed on alternate days from day 8 of the cycle, until the mean maximal diameter (MMD) measured in two planes (sagittal and transverse) of the dominant follicle measured ≥14 mm, after which they were performed daily. Women were asked to use urinary LH kits twice daily (06:00–08:00 and 18:00–20:00) in order to identify the onset of LH surge prior to spontaneous ovulation. An injection of 5,000 IU hCG (Pregnyl (Organon, Cambridge, UK)) was given when the MMD of the lead follicle measured ≥17 mm (17.0–18.1 mm). If the urinary LH kit was positive, ultrasound directed oocyte retrieval (UDOR) was performed the day after the surge was detected; otherwise it was planned for 36 h after hCG. For subsequent analysis, women who had a spontaneous LH surge and women who were administered exogenous hCG were grouped together as the MNC cohort. All women in the COH arm underwent the long protocol. Pituitary downregulation was attained using leuprorelin acetate SR 3.75 mg (Prostap (Wyeth, Maidenhead, Berkshire, UK)) administered on the first day of the menstrual cycle. COH was achieved with 225 IU human menopausal gonadotropin (HMG) daily (Menopur, Ferring, Slough, Berkshire, UK). As with the MNC-IVF/ICSI cycle, when one or more follicles had an MMD of ≥17 mm, 5,000 IU hCG (Pregnyl (Organon, Cambridge, UK)) was administered 36 h prior to UDOR.

All UDORs were performed between 09:00 and 11:00 to accommodate putative circadian variations in ovarian physiology. Whole blood was collected immediately before sedation in EDTA vacutainers. Dead space (containing 1.5 mL 0.9% sodium chloride solution) within the oocyte harvesting needle and tubing was constant/uniform throughout such that the first 1.5 mL aspirated was checked and discarded. Subsequent aspirate was considered to contain FF and, following oocyte assessment and retrieval, was subsequently labelled to ensure longitudinal tracking of the corresponding oocyte to its fate (for multifollicular cycles). Follicles were flushed up to four times with culture medium (Enhance HTF Culture Medium with HEPES; Conception Technologies, San Diego, California, USA) if no oocyte was obtained in the initial aspirate to minimise the risk of inadvertently aspirating a second follicle and collecting the oocyte from the previous one due to being contained within the dead space. All ultrasonically visible follicles were individually aspirated irrespective of their size. The aspiration pressure applied was uniform on all follicles (183–185 mm/Hg).

All samples were immediately stored on ice. Whole blood and FF samples were centrifuged (2,000 rpm at 4°C for 10 minutes) to isolate plasma and remove cell debris, respectively. All samples were frozen at −80°C within one hour of retrieval until required for analysis. In the MNC cohort, only the FF from the single dominant follicle was analysed. In the COH cohort, FF analysis was performed on the follicles yielding the oocytes that generated transferred embryos (since double ETs were performed in these cycles, the follicle selected for analysis was the one yielding the embryo with the highest morphological grading).

In both cycles, routine procedures for fertilization with IVF/ICSI were performed as previously described [64]. Embryo transfer (ET) was performed 84–90 h after hCG injection. A single ET was performed in the MNC cohort, whilst a double ET was performed in the COH cohort (as per ACU protocols at the time). All women in the MNC cohort who underwent an ET received 2,500 IU hCG (Pregnyl) on the day of ET and again 72 h later for luteal support. Women in the COH cohort who developed <15 follicles had an identical luteal support regimen, whereas those women with ≥15 follicles following COH were given daily intramuscular injection of 100 mg progesterone (Gestone, Nordic Pharma, Reading, Berkshire, UK), which was continued throughout the first trimester of pregnancy. Pregnancy tests were performed on first void urine 14 days after ET with a commercial urinary kit. A clinical pregnancy was defined as one demonstrating a gestational sac with a fetal pole and a fetal heart or an ectopic pregnancy by TVUSS at 6-7 weeks' gestation.

### 2.2. Fluid-Phase Multiplex Immunoassay

Cytokine levels in both FF and plasma were measured by fluid-phase cytometric multiplex immunoassay (Bio-Rad Laboratories, Hercules, CA, USA) (Bio-rad assays: Human Cytokine 27-plex Assay M50-0KCAF0Y; Human Cytokine 21-plex Assay MF0-005KMII) using a Luminex 100 cytometer (Luminex Corporation, Austin, Texas, USA) equipped with BioPlex 4.0 Manager software (Bio-Rad Laboratories, Ltd., Hertfordshire, UK), as previously described [65]. Target cytokines included interleukin- (IL-) 1 receptor antagonist (ra), IL-2ra, IL-3, IL-6, IL-7, IL-8, IL-9, IL-10, IL-12 (p40), IL-12 (p70), IL-13, IL-15, IL-16, IL-18, leukaemia inhibitory factor (LIF), granulocyte macrophage-colony stimulating factor (GM-CSF), macrophage- (M-) CSF, granulocyte- (G-) CSF, stem cell factor (SCF), interferon- (IFN-) *α*, IFN-*γ*, IFN-*γ* inducible protein- (IP-10), TNF-*α*, TNF-*β*, TNF related apoptosis inducing ligand (TRAIL), VEGF, platelet derived growth factor (PDGF), basic fibroblast growth factor (b-FGF), nerve growth factor- (NGF-) *β*, stem cell growth factor- (SCGF-) *β*, growth regulated oncogene- (GRO-) *α*, macrophage inflammatory protein- (MIP-) 1*β*, monocyte chemoattractant protein- (MCP-) 1, MCP-3, eotaxin, regulated upon activation of normal T cell expressed and secreted (RANTES), stromal cell-derived factor- (SDF-) 1*α*, cutaneous T-cell attracting chemokine (CTACK), monokine induced by IFN-*γ* (MIG), and macrophage migration inhibitory factor (MIF).

### 2.3. Contamination and Standardisation

Oocyte retrieval frequently results in disruption of the intraovarian vasculature such that blood (macroscopic or microscopic) contaminates the FF retrieved [66]. Furthermore, although the needle and tubing were primed with normal saline at the commencement of aspiration of each ovary, in between subsequent follicular aspirations, protein-free flush medium formulated with gentamicin (enhanced HTF culture medium with HEPES; Conception Technologies, San Diego, California, USA) was used, with potential dilution of the FF [67]. Such contamination/dilution was accounted for in the FF cytokine analysis. This entailed cytokine, total protein (by Lowry assay, Bio-Rad), and von Willebrand factor (vWF; by enzyme-linked immunosorbent assay; R&D Systems, Abingdon, UK) measurement in both plasma and FF. Since vWF is a large plasma multimeric glycoprotein that does not pass through the basement membrane and is not produced by follicular cells, it enabled accurate quantification of FF blood (and therefore circulatory cytokine) contamination (present authors, manuscript under review). The dilutional effect of the flush medium was instead accounted for by standardising both FF and plasma (the latter to enable a valid comparison with the former) cytokine concentrations to total protein (pg cytokine/mg protein).

### 2.4. Statistical Analysis

Chi-squared, independent samples *t*-tests, or Mann-Whitney *U* tests were used to compare MNC and COH patient demographics, cycle details, FF, and plasma cytokines following tests for normal distribution by Shapiro-Wilk test (Stata/SE 11.1, Texas, USA). In order to address the interrelationships between multiple cytokines and the impact that COH has upon these, heat maps were generated using R 2.7.0 software (R Foundation for Statistical Computing, Vienna, Austria). Correlations between the different cytokines were determined for MNC and COH data using Kendall's tau as a measure of correlation (Stata/SE 11.1). Resulting *P* values were adjusted for multiple comparisons with Holm's correction (*P* < 0.05 was considered significant).

## 3. Results and Discussion 

### 3.1. Participant, MNC, and COH Cycle Demographics

In the MNC cohort, one patient had a positive LH surge and therefore did not receive exogenous hCG. At the time of UDOR 12 hours after the positive surge, spontaneous ovulation had occurred such that no FF was retrieved and an oocyte was not obtained. This patient was therefore excluded from the study. No statistically significant differences were noted in patient demographics (age, BMI, baseline endocrine profile (FSH, LH, and E_2_), and ethnicity), day of UDOR, follicular aspirate volume, oocyte maturity, and cycle outcome between the two groups (Table 1).

### 3.2. Follicular Fluid and Plasma Cytokines

**Figure 1:**
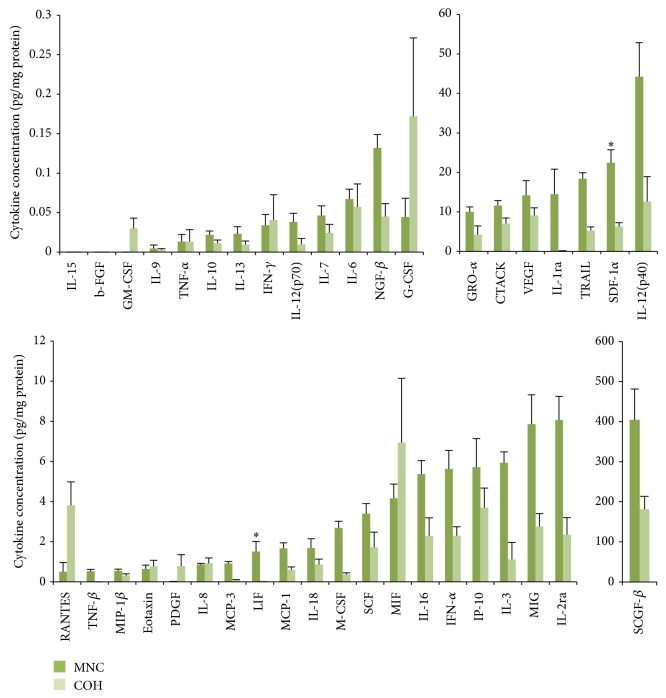
Mean (± SEM) FF cytokine concentrations in MNC (*n* = 9) and COH (*n* = 10) cycles (^*^significant difference from COH cycle; *P* < 0.05).

**Figure 2:**
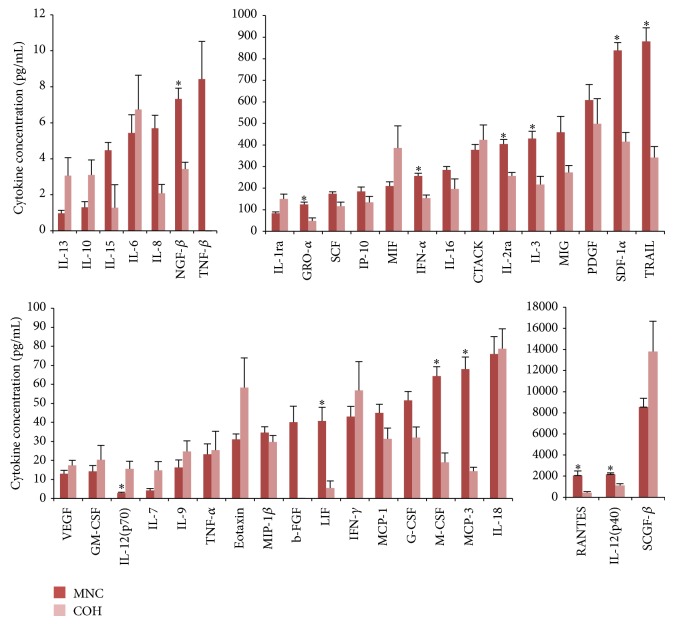
Mean (± SEM) plasma cytokine concentrations in MNC (*n* = 9) and COH (*n* = 10) cycles (^*^significant difference from COH cycle; *P* < 0.05).

Most FF cytokines (29 out of 40) were found to be at higher concentrations in the MNC group compared to the COH group (binomial test: *P* < 0.001). This relationship was statistically significant for LIF (*P* < 0.01) and SDF-1*α* (*P* < 0.05) (Figure 1). As with FF, the majority of circulatory cytokines in the MNC cohort were present at higher concentrations than in the COH group (Figure 2), a relationship which was significant for 12 of these: IL-2ra, IL-3, IL-12 (p40), LIF, M-CSF, IFN-*α*, TRAIL, NGF-*β*, GRO-*α*, MCP-3, RANTES, and SDF-1*α* (*P* < 0.05). Conversely, plasma IL-12 (p70) levels were present at significantly higher levels following COH (*P* < 0.05) (Figure 2).

**Figure 3:**
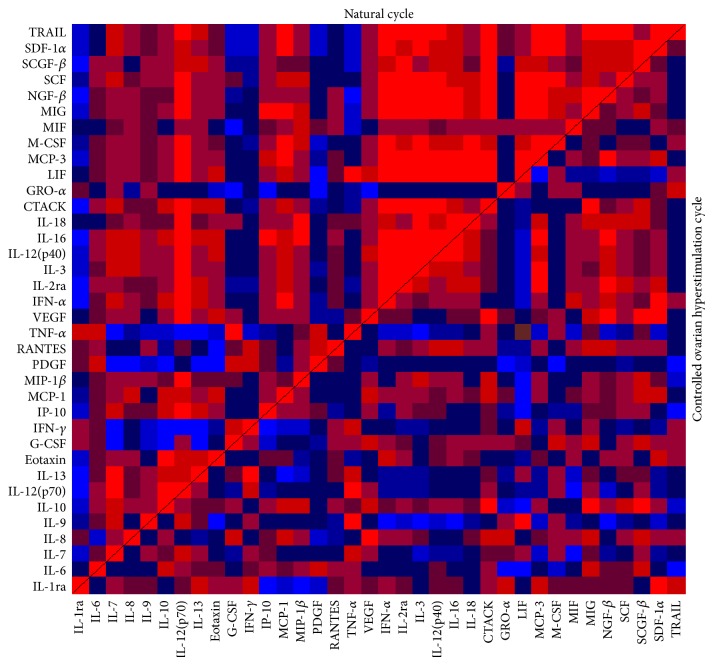
Heat maps demonstrating correlations between cytokines in periovulatory FF in MNC and COH cycles. Each square represents the correlation between specific cytokines: a red square represents a significantly positive correlation whilst a blue square represents a significantly negative correlation. A black square represents no significant relationship and the darker shades of red and blue represent weaker positive and negative correlations, respectively.

Heat maps were generated following correlation analysis using Kendall's tau to demonstrate FF cytokine interrelationships (Figure 3). Significantly more pairs of cytokines exhibited strong correlations in the MNC data compared to the COH data (binomial test) (*P* < 0.001). Various relationship alterations were also noted; for example, LIF and TNF-*α* demonstrated a weak negative correlation in the MNC group (Kendall's tau: −0.08) compared with a strong positive correlation following COH (Kendall's tau: 0.46). When plasma:FF cytokine ratios were compared between the MNC and COH cohorts, no statistically significant differences were identified.

Gonadotropin use in COH has previously been recognised to perturb the intrauterine cytokine milieu. Significantly higher concentrations of various cytokines including IL-1*β*, IL-5, IL-10, IL-12, IL-17, TNF-*α*, and eotaxin have been recorded in endometrial secretions from stimulated cycles compared to NCs [68]. Similarly, in the ovary, it has been suggested that exogenous gonadotropins may influence the levels of cytokines such as IL-1*β*, IL-6, and TNF-*α* following earlier studies on FF [69]. More recently, de Los Santos et al. [70] demonstrated altered cumulus cell gene expression for leukocyte differentiation and T-cell activation and regulation following COH, which may in turn influence follicular cytokine profiles as highlighted by the present findings.

To the best of our knowledge, FF has not previously been analysed for such an extensive range of cytokines in NC/MNC and COH cycles. In the present pilot study, FF concentrations of 29 out of the 40 cytokines analysed were found to be higher in the MNC cohort, significantly so for LIF and SDF-1*α*. LIF is an embryotrophic cytokine whose secretion by GCs and stromal cells into FF has previously been shown to be stimulated by hCG [71–73]. Since both the MNC and COH cohorts in this study received identical doses of exogenous hCG, the elevated levels of LIF in MNC FF may represent an enhanced response to hCG, whereas the likely perturbed cytokine response allied to COH may reflect a reduced sensitivity to hCG resulting in lower LIF concentrations. COH also appeared to perturb relationships between cytokines, highlighted by the relative changes in intrafollicular LIF and TNF-*α* (where the latter modulates ovarian stromal cell secretion of the former) [71]. The heat map displays a strong positive correlation between LIF and TNF-*α* in the MNC cohort whereas this relationship is weakened following COH. Interestingly, FF LIF concentrations have also been correlated with E_2_ concentrations (possibly through its role in enhancing aromatase expression) which, in turn, relate to follicular maturity [74, 75]. Although a comparable causal association has not been identified to date in the ovary, 17-*β* oestradiol is known to induce LIF synthesis in bovine oviduct epithelial cells [76]. It is tempting to speculate that an analogous mechanism is at play in the ovary, where the supraphysiological E_2_ levels associated with COH may impair the induction of follicular LIF synthesis, with a consequent impact on the FF milieu and oocyte quality.

SDF-1*α* is a chemokine secreted by oocytes, which acts in a paracrine manner to prevent follicular activation, thereby controlling the entry of primordial follicles into the growing pool in NCs [77]. Furthermore, FF SDF-1*α* has previously been positively correlated with FF VEGF levels in COH cycles, where it is believed to play a proangiogenic role in supporting follicular growth [78]. Our findings corroborate this correlation in the COH group (Figure 3). By contrast, this relationship was much weaker in the MNC cohort, suggesting that it may thus in part be gonadotropin dependent.

Akin to what was noted for FF, plasma cytokine levels in the MNC group were higher than in COH cycles. The exception was circulatory IL-12 (p70), which was measured at significantly higher concentrations following COH. Furthermore, correlations between FF IL-12 (p70) and other cytokines were markedly altered following stimulation. In the MNC cohort, most FF cytokines were positively correlated with IL-12 (p70), whilst this relationship was reversed following COH. Conversely, IFN-*γ* and TNF-*α* were negatively correlated with IL-12 (p70) in the MNC group and positively in the COH cohort. Interestingly, an intricate triumvirate relationship between these particular cytokines has been identified in the regulation of inflammatory responses. IL-12 (p70) induces IFN-*γ* production [79] and, in turn, IFN-*γ* markedly augments IL-12 (p70) production, thereby providing a key inflammatory amplifying mechanism [80]. By contrast, TNF-*α* is thought to inhibit IFN-*γ*-induced IL-12 (p70) production, as demonstrated by Hodge-Dufour et al. [81], whose TNF^+/+^ mice exhibited a prompt inflammatory response which resolved spontaneously compared with a delayed, more vigorous, inflammatory response leading to death associated with elevated IL-12 levels in TNF^−/−^ mice when injected with* Corynebacterium parvum*. Thus, TNF-*α* is thought to contribute to the resolution of IL-12- (p70-) driven inflammatory processes. In the MNC cohort, TNF-*α* was negatively correlated with both IL-12 (p70) and IFN-*γ*, thus suggesting that, in the absence of stimulatory gonadotropins, TNF-*α* of follicular cell origin may play an analogous role via its capacity to regulate IL-12 (p70) production. A positive correlation between these FF cytokines in combination with significantly elevated systemic IL-12 (p70) levels suggests a disruption of this mechanism following COH. An analogous disruption in the ovary following COH could thus contribute to the detrimental sequelae of increased IL-12 (p70) and IFN-*γ* levels despite the observed compensatory rise in modulatory TNF-*α* levels.

Fluctuations in systemic white cell populations have been attributed to stimulation with exogenous gonadotropins such that the total number of circulatory leukocytes and neutrophils was increased on the day of hCG administration in COH compared to NCs [82]. Other studies have reported an increase in plasma white cell count and G-CSF, but not in M-CSF or IL-6, during COH [83]. Furthermore, various cytokines have been found to be elevated in FF following COH, including IL-1*β*, IL-6, and TNF-*α* [69]. The main difference between the present study and those cited is our administration of exogenous hCG in the MNC cohort. Both LH and hCG induce IL-1*β* and TNF-*α*, both of which subsequently upregulate GM-CSF expression [38]. Despite identical doses of hCG being used to trigger ovulation in both groups in the current study, GM-CSF in particular was measured at higher (although not statistically significant) levels following COH in both FF and plasma. Furthermore, following COH, there was a trend towards several other proinflammatory cytokines in both FF and plasma to be found at higher concentration, with IL-12 (p70) levels in particular being significantly higher in COH plasma samples. This provides further evidence that COH can induce both local and systemic inflammatory network deregulations featuring perturbations in cytokine interrelationships which, speculatively, may potentially also impact oocyte viability and treatment outcome. Unfortunately, the present pilot study was not powered to answer this particular question.

## 4. Conclusions

In conclusion, this investigation demonstrated that COH not only alters FF cytokine profiles compared to MNCs but also perturbs their circulatory levels and disrupts their interrelationships. Given their central role in orchestrating normal follicular physiology, these changes have the potential to adversely affect follicular function and compromise oocyte viability.

## Figures and Tables

**Table 1:** Participant and cycle demographics in MNC and COH cycles.

	NC Cycle	COH Cycle	*P*-value
Age (years)	30.8 ± 0.72 (27–34)	31.9 ± 1.20 (24–35)	0.21
BMI (kg/m^2^)	23.5 ± 0.86 (20.0–28.0)	24.0 ± 0.87 (20.0–30.0)	0.88
Baseline FSH (IU/L)	5.2 ± 0.40 (4.0–8.0)	6.0 ± 0.45 (3.9–7.9)	0.24
Baseline LH (IU/L)	5.2 ± 0.48 (2.6–7.1)	4.6 ± 0.55 (1.5–7.4)	0.44
Baseline E_2_ (pmol/L)	115.2 ± 12.23 (73–162)	117.8 ± 18.14 (25–193)	0.92
Day of cycle for aspiration	14 ± 0.93 (10–20)	13.1 ± 0.34 (12–15)	0.35
Follicular volume (mL)	2.3 ± 0.40 (0.5–4.0)	3.0 ± 0.51 (1.0–6.5)	0.62
Mature oocyte (%)	70^a^	100^b^	0.06
Clinical pregnancy (%)	20^c^	30^c^	0.61

Mean ± SEM (range), unless otherwise specified. ^a^One patient spontaneously ovulated, therefore 9 oocytes were retrieved; ^b^Follicles yielding a mature oocyte which was subsequently transferred as an embryo were included in this cohort; ^c^Clinical pregnancy rate calculated per embryos transferred [MNC: 7 embryos transferred; COH; 10 embryos transferred].
